# Shear induced collateral artery growth modulated by endoglin but not by ALK1

**DOI:** 10.1111/j.1582-4934.2012.01561.x

**Published:** 2012-09-26

**Authors:** Leonard Seghers, Margreet R de Vries, Evangelia Pardali, Imo E Hoefer, Beerend P Hierck, Peter ten ten Dijke, Marie Jose Goumans, Paul HA Quax

**Affiliations:** a Department of Surgery, Leiden University Medical CenterLeiden, The Netherlands; b Department of Physiology Institute for Cardiovascular Research, VU University Medical CenterAmsterdam, The Netherlands; c Laboratory of Experimental Cardiology, University Medical Center UtrechtUtrecht, The Netherlands; d Department of Anatomy and Embryology, Leiden University Medical CenterLeiden, The Netherlands; e Department of Molecular Cell Biology, Leiden University Medical CenterLeiden, The Netherlands; f Einthoven Laboratory for Experimental Vascular Medicine, Leiden University Medical CenterLeiden, The Netherlands

**Keywords:** arteriogenesis, angiogenesis, TGF-beta, ischaemia, shear stress

## Abstract

Transforming growth factor-beta (TGF-β) stimulates both ischaemia induced angiogenesis and shear stress induced arteriogenesis by signalling through different receptors. How these receptors are involved in both these processes of blood flow recovery is not entirely clear. In this study the role of TGF-β receptors 1 and endoglin is assessed in neovascularization in mice. Unilateral femoral artery ligation was performed in mice heterozygous for either endoglin or ALK1 and in littermate controls. Compared with littermate controls, blood flow recovery, monitored by laser Doppler perfusion imaging, was significantly hampered by maximal 40% in endoglin heterozygous mice and by maximal 49% in ALK1 heterozygous mice. Collateral artery size was significantly reduced in endoglin heterozygous mice compared with controls but not in ALK1 heterozygous mice. Capillary density in ischaemic calf muscles was unaffected, but capillaries from endoglin and ALK1 heterozygous mice were significantly larger when compared with controls. To provide mechanistic evidence for the differential role of endoglin and ALK1 in shear induced or ischaemia induced neovascularization, murine endothelial cells were exposed to shear stress *in vitro*. This induced increased levels of endoglin mRNA but not ALK1. In this study it is demonstrated that both endoglin and ALK1 facilitate blood flow recovery. Importantly, endoglin contributes to both shear induced collateral artery growth and to ischaemia induced angiogenesis, whereas ALK1 is only involved in ischaemia induced angiogenesis.

## Introduction

In human adult life neovascularization comprises two different processes, angiogenesis, in which ischaemia induced sprouting of vessels leads to the formation of new capillaries, and arteriogenesis, where shear-stress-driven maturation of pre-existing collateral arteries results in formation of mature collateral arteries. Whereas angiogenesis is mainly ischaemia driven, arteriogenesis is a highly inflammation driven process. Next to specific inflammatory cell subsets [1–5], various cytokines and growth factors contribute to arteriogenesis.

Therapeutic application of cytokines, such as monocyte chemoattractant protein and growth factors, such as basic fibroblast growth factor and vascular endothelial growth factor (VEGF) stimulate arteriogenesis [6]. More recently, it was demonstrated that local delivery of TGF-β also stimulates arteriogenesis [7, 8].

Transforming growth factor-beta is a multi-functional cytokine involved in the control of cell division, differentiation, migration, adhesion and programmed cell death. Furthermore, TGF-β is known to mediate inflammation and contributes to vascular remodelling, for example after vessel wall injury [9] and in stimulating blood flow recovery [7]. Moreover, TGF-β is also thought to play a crucial role in the regulation of angiogenesis, especially in tumour angiogenesis [10–12]. TGF-β signalling is initiated by the formation of a membrane complex of two TGF-β type II receptors and two TGF-β type I receptors, also termed activin receptor-like kinases or ALKs. This complex then initiates signalling responses into the cytoplasm by phosphorylating intracellular transcription factors called SMADs [13, 14].

In endothelial cells (ECs), TGF-β signalling is mediated by two TGF-β type I receptors, ALK5, which is also expressed in many other cell types, and ALK1, which is mainly expressed in ECs [15]. In addition, ECs express an accessory TGF-β type III receptor named endoglin (Eng), which is also expressed by vascular smooth muscle cells (SMCs) [16]. Vessel remodelling, EC proliferation and migration are inhibited by TGF-β signalling *via* TβRII/ALK5, which mediates SMAD2/3 phosphorylation [10, 17], and these processes can be stimulated in ECs by TGF-β signalling *via* an Endoglin/TβRII/ALK1 complex which induces SMAD1/5 activation [18, 19]. Endoglin promotes the TGF-β/ALK1 signal transduction pathway [20], whereas ALK1 inhibits TGF-β/ALK5 signalling [12, 21].

Interestingly, ALK1 and its ligand bone morphogenetic protein (BMP) 9 play a dual role in angiogenesis, as both have been reported to inhibit endothelial cell migration [22] and induce vascular quiescence [23], whereas in another study the BMP9/ALK1 cascade has been shown to induce angiogenesis and stimulate endothelial cell proliferation [24]. These observations imply that TGF-β signalling affects vessel formation, which is concentration- and context-dependent, and will be different in the activation and maturation phase of angiogenesis [25].

Both ALK1 and endoglin modulate neovascularization by induction of blood flow recovery. Shear stress is an essential factor in neovascularization and has been demonstrated to play a role in activation of ALK5/TGF-β signalling in the endothelium [26, 27]. Furthermore, these hemodynamic changes are suggested to induce ALK1 expression in ECs [28], and an active role for both endoglin and ALK1 is suggested in shear stress driven vascular adaptation [29, 30]. This suggests that an increase in shear stress during arteriogenesis might modulate the expression of ALK1 and endoglin. Endoglin is involved in the production of TGF-β and VEGF by ECs, the modulation of EC proliferation and differentiation, and recruitment and differentiation of vascular SMCs [31, 32]. After myocardial infarction, endoglin is up-regulated in angiogenic vessels from human and murine hearts [33]. ALK1 also plays an important role in EC proliferation [12] and migration, and stabilization of vessel structure integrity by stimulating recruitment and differentiation of SMCs [28]. In humans deregulated TGF-β signalling by haplo-insufficiency for either endoglin or ALK1 results in arteriovenous malformations that are a hallmark of hereditary haemorrhagic telangiectasia type 1 and type 2 (HHT1 or 2) [19].

The individual role of endoglin and ALK1 in vessel growth is well described, it is yet unknown how these receptors are involved in blood flow recovery after induction of hind limb ischaemia. Jerkic *et al*. [32] studied the effect of haplo-insufficiency for endoglin on capillary angiogenesis in the adductor thigh after ligation of the femoral artery, and demonstrated reduced blood flow and capillary angiogenesis in the upper leg. In this study the analysis was restricted to neovascularization in the adductor thigh muscle only, whereas capillary formation in the ischaemic gastrocnemius muscle was not studied. This is, however, quite important especially in comparison with the effect of ALK1 haplo-insufficiency.

In this study, we investigate the role of endoglin and ALK1 in both shear induced collateral artery growth and ischaemia induced angiogenesis. Induction of hind limb ischaemia in mice heterozygous for either endoglin or ALK1 is a good model to study the involvement of these two receptors in neovascularization.

## Methods

### Mice

All animal experiments were approved by the committee on animal welfare of our institute. For all experiments male mice were used, aged 12–16 weeks. Endoglin (C57BL/6 background; eight backcrosses) heterozygous mice (Eng^+/−^) [34] and ALK1 (C57BL/6; 13 backcrosses) heterozygous mice (ALK1^+/−^ [19] and corresponding littermates were bred in our institute. In both endoglin and ALK1 breeding colonies, mice were genotyped by PCR to detect heterozygosity for either endoglin or ALK1. Wild type littermates were used as control. All animals were fed regular chow diet (Sniff Spezialdiäten GMBH, Soest, Germany).

### Surgical procedure and analysis of arteriogenesis

Surgical induction of hind limb ischaemia in the mice, as well as analysis of collateral formation by either angiography or laser-Doppler-perfusion-imaging (LDPI; Moor Ltd, Axminster, UK), and tissue collection was performed as described [4, 35]. Briefly, hind limb ischaemia was induced by electro-coagulation of the left femoral artery, and the right leg was used as an internal control.

### Angiography

To study collateral vessel development, post-mortem angiograms of both hind limbs were made using polyacrylamide-bismuth contrast mixture (Sigma-Aldrich Chemie, Zwijndrecht, The Netherlands) 7 days after femoral artery occlusion, as described [35]. Grading of collateral filling was performed in a single blinded fashion by two independent observers and was based on the Rentrop score. Grading was as follows: 0 = no filling of collaterals, 1 = filling of collaterals only, 2 = partial filling of distal femoral artery, 3 = complete filling of distal femoral artery.

### Tissue handling

Adductor thigh and gastrocnemius muscles were dissected upon killing of the mice. Adductor thigh muscles were dissected by cutting the tendons from the distal and medial part of the femur and at the os pubis. The gastrocnemius muscle was dissected by cutting the calcaneal tendon and the tendons at the distal femur. Subsequently muscles were formalin-fixated (4%) overnight at room temperature. The collateral artery zone was respected by transversal transection of the adductor thigh muscles at the centre and subsequent direct embedding with the section plane in upright position. Gastrocnemius muscles were treated likewise.

### Immunohistochemistry and analysis

Five μm thick paraffin-embedded cross sections were stained using antibodies for PECAM-1 (CD31) (BD Biosciences, San Jose, CA, USA), α-smooth muscle (αSM)-actin (Clone1A4) (DAKO, Glostrup, Denmark), Erythroid lineage TER-119 (Santa Cruz Biotechnology, Santa Cruz, CA, USA) for erythrocyte staining, Ki-67 staining using the NCL-L-Ki67-MM1 antibody (Novocastra Reagents, Valkenswaard, The Netherlands) for proliferation (EDTA antigen retrieval) or Masson Trichrome kit HT15 for Masson Trichrome staining (according to manufacturer's protocol; Sigma-Aldrich Chemie BV). Muscles stained for both CD31 and αSM-actin were quantified from sections photographed randomly (three images per section, nine representative images per muscle per mouse) using image analysis (Qwin; Leica, Wetzlar, Germany), and this was all performed in a single blinded observer fashion. During analysis arteries directly accompanied by a vein are considered main arteries and excluded from measurement. Furthermore, in the case of measuring ellipse shaped SMA positive vessels that can be appreciated to be sectioned on an angle, the area is calculated based on the short axis diameter to prevent from overestimated vessel diameters.

### Endothelial cell culture

Mouse embryonic endothelial cell (MEEC) isolation was previously described [36]. Cells were passaged twice a week and maintained on 1% w/v gelatin (Merck, Darmstadt, Germany) in DMEM medium (Invitrogen, Breda, the Netherlands) supplemented with 4.5 g/l d-glucose (Invitrogen), 25 mM Hepes (Invitrogen), 110 mg/l sodium pyruvate (Invitrogen), 10% (v/v) heat inactivated Fetal Calf Serum (Sigma-Aldrich Chemie, Steinheim, Germany), 1% (v/v) antibiotic/antimyotic solution (Invitrogen) and 2 mM l-glutamine (Invitrogen). For the shear stress experiments, MEEC were seeded on fixed 1% (w/v) gelatin coated cover slips and grown to confluence. EC were subjected to 0.5 Pa or -5 dyn/cm^2^ shear stress for 24 hrs at 37°C and 5% CO_2_ in a re-circulation parallel plate flow system, as previously reported [27]. Responses of shear-exposed cells were compared with those of static cultures. Directly following exposure to flow cells were lysed for RNA isolation.

### mRNA analysis

#### Q-PCR

Total RNA was isolated (RNeasy; Qiagen Benelux, Venlo, The Netherlands) and was treated with DNAse-I (Qiagen) according to the manufacturer's protocol. IScript cDNA synthesis kit (Bio-Rad, Veenendaal, The Netherlands) was used to reverse transcribe 500 ng of RNA into cDNA. Real-time Q-PCR was performed with iQ SYBR Green Supermix (Bio-rad) in a Mx3000 real-time thermocycler (Stratagene, Santa Clara, CA, USA), as described [37]. The reaction mixture consisted of the following: 1x PCR Master Mix, 1 μl cDNA template and 10 pmol of each specific primer. Dissociation analysis was performed in all reactions to exclude the presence of primer-dimers and confirm the amplification of unique targets. No template controls were used as negative controls. Relative expression levels were normalized to the housekeeping gene beta-actin (acbt) to compensate for the differences in RNA input.

### Statistical analysis

Results are expressed as mean ± S.E.M. Comparisons between medians were performed using the Mann–Whitney or Wilcoxon test, as appropriate. Ordinal scores (e.g. angiography) were compared using Pearson Chi-Square test. A *P*-value <0.05 was considered statistically significant.

## Results

### Delayed blood flow recovery and reduced collateral artery size in Eng^+/−^ mice

**Fig 1:**
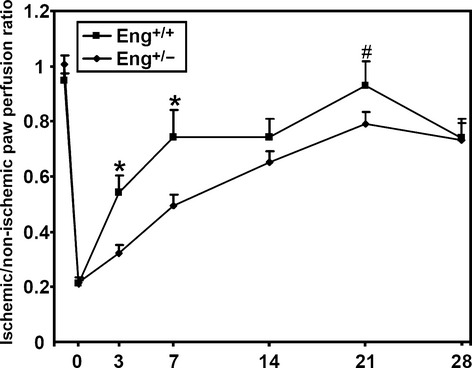
Ischemic/non-ischaemic paw perfusion ratios, measured by Laser Doppler Perfusion Imaging (LDPI) over 28 days of endoglin heterozygous (Eng^+/−^, *n* = 10) and control mice (Eng^+/+^, *n* = 9; originally 10, but 1 mice died as a result of an unrelated cause of death and was therefore not included in the analysis). (*P-value <0.05), (#*P*-value = 0.05).

After induction of hind limb ischaemia by femoral artery ligation Eng^+/−^ mice revealed a significant delay in blood flow recovery when compared with controls. Blood flow recovery was significantly reduced by 40% after 3 days, 33% after 7 days and by 16% after 21 days (*P*-value = 0.05). Eng^+/−^ mice reached a maximum paw perfusion ratio at 28 days of 0.73 ± 0.06 (Fig. 1).

**Fig 2:**
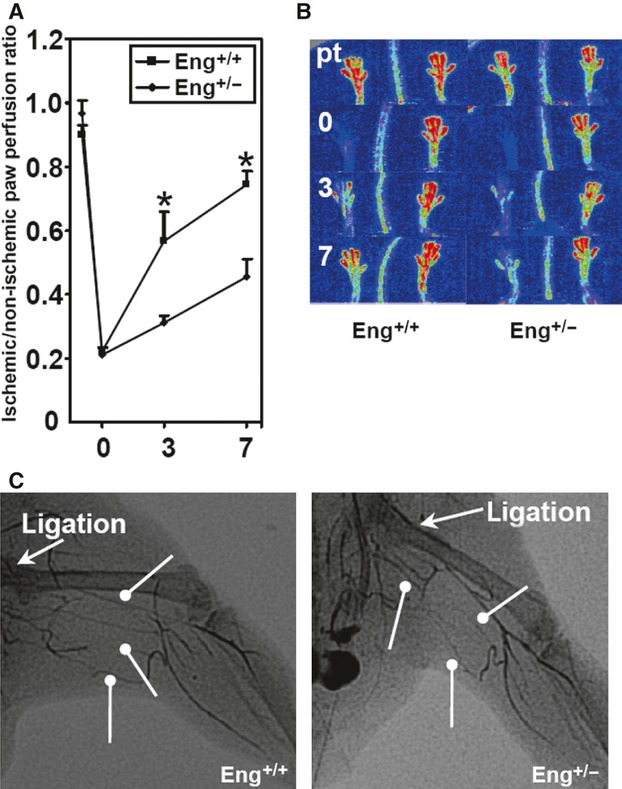
(A) Ischemic/non-ischaemic paw perfusion ratios of Eng^+/−^ (*n* = 9; originally 10, but 1 mice died as a result of an unrelated cause of death and was therefore not included in the analysis) and control mice (*n* = 8; originally 10, but 2 mice died as a result of an unrelated cause of death and were therefore not included in the analysis), measured by LDPI over 7 days. (*P-value <0.01) (B) Representatives of LDPI images of Eng^+/−^ and control mice that were used to obtain post-mortem angiographic images. Several time-points are displayed: pre-treatment (pt), *t* = 0, *t* = 3 and *t* = 7. (C) Representative angiographic images of upper hind limb of Eng^+/−^ and control mice 7 days after femoral artery ligation. Arrow indicates site of femoral artery ligation. Rods indicate collateral arteries filled with contrast agent.

For performing post-mortem angiography to demonstrate presence of collateral arteries in adductor thigh muscle, Eng^+/−^ mice (*n* = 10) and controls (*n* = 10) were killed 7 days after femoral artery ligation. LDPI follow-up until 7 days showed significant impaired blood flow recovery in Eng^+/−^ mice when compared with controls at 3 and 7 days after ligation (Fig. 2A and B), similarly as was observed in the long-term follow-up experiment demonstrated in Fig. 1. As shown by representative angiographic images (Fig. 2C), no obvious differences in the presence of collaterals between Eng^+/−^ mice and controls could be observed.

**Fig 3:**
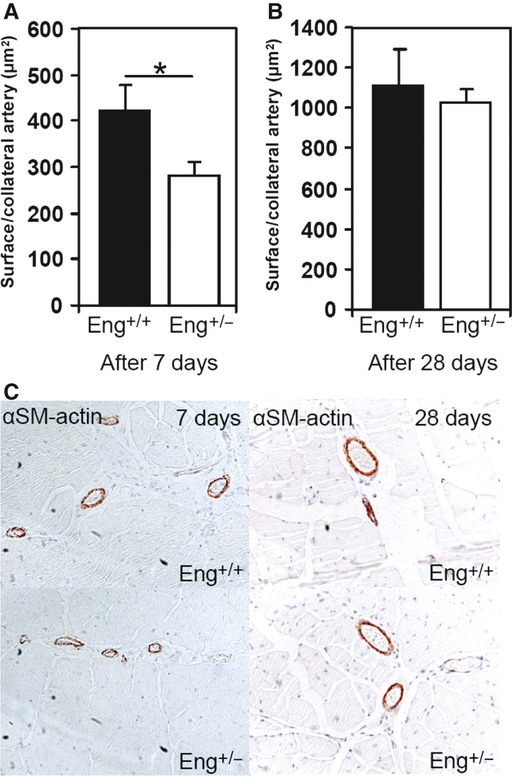
Analysis for collateral artery size, measured as surface (μm^2^) per collateral artery in left adductor thigh muscles of Eng^+/−^ (open bars) and control mice (closed bars) (*n* = 6–10) (A) 7 days and (B) 28 days after femoral artery ligation. (*P-value <0.05) (C) Representative pictures (magnification 15×) of SMC-a stained adductor thigh muscles of Eng^+/−^ and control mice at 7 and 28 days after femoral artery ligation.

In the search for an explanation for the delay in blood flow recovery, we determined whether collateral artery size was affected in Eng^+/−^ mice and performed an αSM-actin staining on adductor thigh muscles collected 7 days after ligation. The analysis for collateral artery size revealed significant smaller collateral arteries in Eng^+/−^ mice when compared with controls, monitored as collateral artery surface on cross-sections (Fig. 3A). Collateral artery size in adductor thigh muscles collected 28 days after ligation was not different between Eng^+/−^ and control mice (Fig. 3B). Figure 3C depicts representative pictures of αSM-actin staining in adductor thigh muscles at both time-points. A Masson Trichrome staining, performed 7 days after femoral artery ligation, demonstrated abundant collagen deposition in left challenged adductor thigh muscles of endoglin heterozygous mice when compared with control mice (Supplemental Fig. S1A). To see whether endoglin was expressed around blood vessels in the adductor thigh muscles after femoral artery ligation, challenged left adductor thigh muscles and control muscles were stained for endoglin, which revealed that endoglin was expressed by blood vessels in control mice and that this was reduced in endoglin heterozygous mice (Supplemental Fig. S2).

### Enlarged capillaries in Eng^+/−^ mice after induction of ischaemia

**Fig 4:**
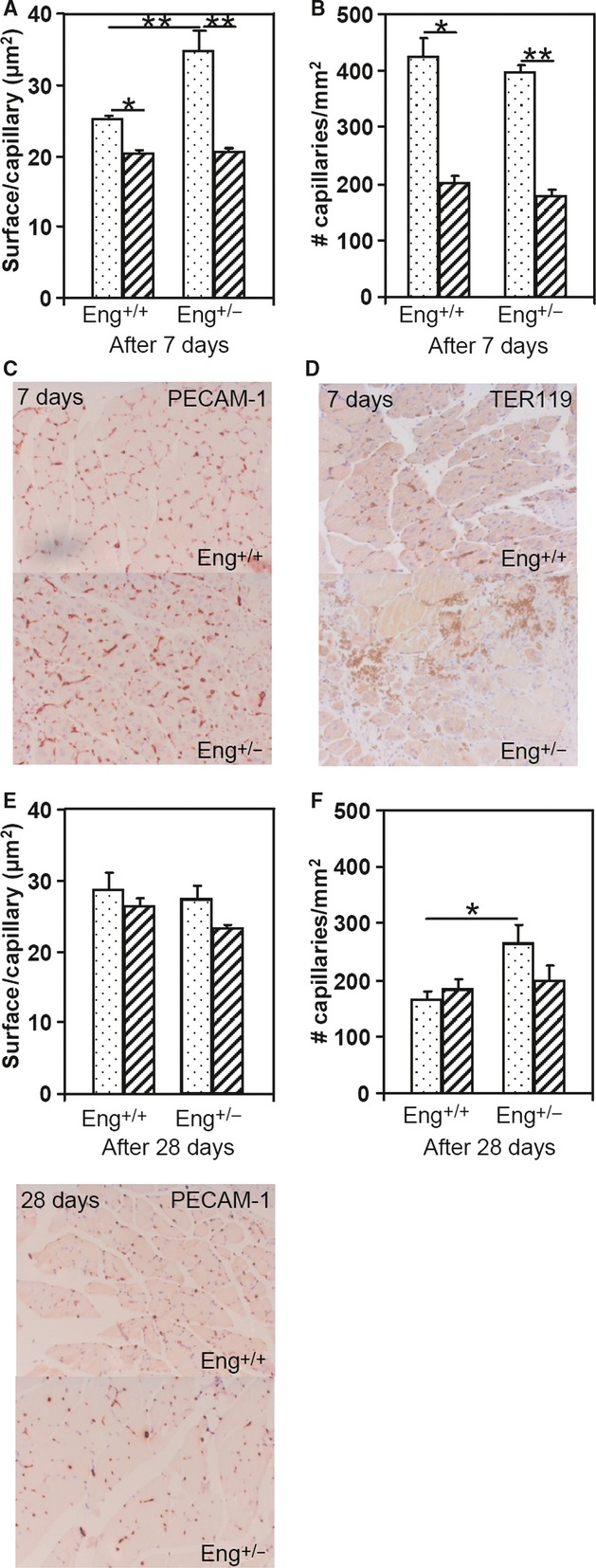
Quantification of ischaemia induced angiogenesis by analysis of PECAM-1 stained left (ligated-side) and right (control-leg) calf muscles of Eng^+/−^ and control mice at 7 and 28 days after femoral artery ligation. Dotted bars represent left *P*-calf muscles, dashed bars represent right calf muscles. (*P-value <0.05, ** *P*-value <0.01) (A) Capillary size, measured as surface per individual capillary (μm^2^), 7 days after femoral artery ligation (*n* = 8–9 per group). (B) Capillary density, measured as number of capillaries per mm^2^, 7 days after femoral artery ligation. (*n* = 8–9 per group). (C) Representative pictures (magnification 20×) of PECAM-1 stained ischaemic calf muscles of Eng^+/−^ and control mice at 7 days after femoral artery ligation. (D) Representative pictures (magnification 20×) of TER-119 staining (erythrocytes) demonstrating evasion of erythrocytes outside of the capillary vasculature into the interstitial tissue compartment in Eng^+/−^ ischaemic calf muscles when compared with ischaemic calf muscles of control mice 7 days after femoral artery ligation. (E) Capillary size, measured as surface per individual capillary (μm^2^), 28 days after femoral artery ligation (*n* = 9–10 per group). (F) Capillary density, measured as number of capillaries per mm^2^, 28 days after femoral artery ligation (*n* = 9–10 per group). (G) Representative pictures (magnification 20×) of PECAM-1 stained ischaemic calf muscles of Eng^+/−^ and control mice at 28 days after femoral artery ligation.

To analyse whether endoglin deficiency also affected the angiogenic response in the ischaemic hind limb, the calf muscles (gastrocnemius muscle) were analysed and quantification of angiogenesis was performed by analysing the capillaries formed in challenged (left) and unchallenged (right) calf muscles, 7 days and 28 days after ligation (PECAM-1 staining). As depicted in Fig. 4A, analysis for capillary size demonstrated significant larger capillaries in Eng^+/−^ mice than observed in controls, 7 days after ligation. In both Eng^+/−^ and control mice a significant increase in capillary density is observed, 2.2 and 2.1-fold increase respectively, when compared with capillary density in right calf muscles. No difference in capillary density was observed between both groups (Fig. 4B), which is also shown in the representative images in Fig. 4C. To monitor for capillary dysfunction, e.g. leaky capillaries by reduced integrity of the endothelium, by a TER-119 staining for erythrocytes, revealed evasion of erythrocytes outside of the capillary vasculature into the interstitial tissue compartment in ischaemic calf muscles of Eng^+/−^ mice 7 days after induction of hind limb ischaemia. Whereas in the wild type mice no erythrocyte extravasation could be observed, in 7 of 10 of the Eng^+/−^ mice erythrocyte extravasation could be observed.

Analysis for capillary size in calf muscles collected 28 days after ligation revealed a reduction in capillary size in the challenged left calf muscles of Eng^+/−^ mice compared with the capillary size observed at day 7. After 28 days capillary size was not different anymore between Eng^+/−^ mice and controls (Fig. 4E). A similar pattern was observed for capillary density, as capillary density in challenged left calf muscles of both Eng^+/−^ mice and controls was decreased when compared with capillary densities observed 7 days after femoral artery ligation in left calf muscles of both Eng^+/−^ mice and controls. As depicted in Fig. 4F and G, this decrease was less pronounced in Eng^+/−^ mice that displayed significant higher capillary densities in left calf muscles when compared with controls.

### Delayed blood flow recovery in ALK1^+/−^ mice

**Fig 5:**
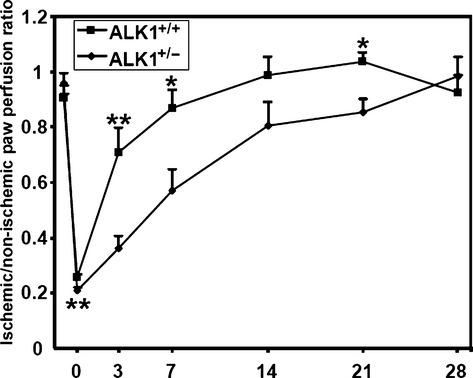
Ischemic/non-ischaemic paw perfusion ratios of ALK1 heterozygous (ALK^+/−^, *n* = 10) and control mice, measured by LDPI over 28 days (*n* = 9; originally 10, but 1 mice died as a result of an unrelated cause of death and was therefore not included in the analysis) (*P-value <0.05, ** *P*-value <0.01).

After induction of hind limb ischaemia by femoral artery ligation in ALK1^+/−^ mice, blood flow recovery was significantly delayed when compared with controls. Directly after femoral artery ligation, blood flow recovery in ALK1^+/−^ mice was significantly reduced by 20% and was also significantly reduced after 3, 7 and 21 days by 49%, 33% and 17% respectively (Fig. 5). Despite this delay in blood flow recovery, ALK1^+/−^ mice reached a paw perfusion ratio of 0.98 ± 0.07 at 28 days which was equal to that observed in control mice.

**Fig 6:**
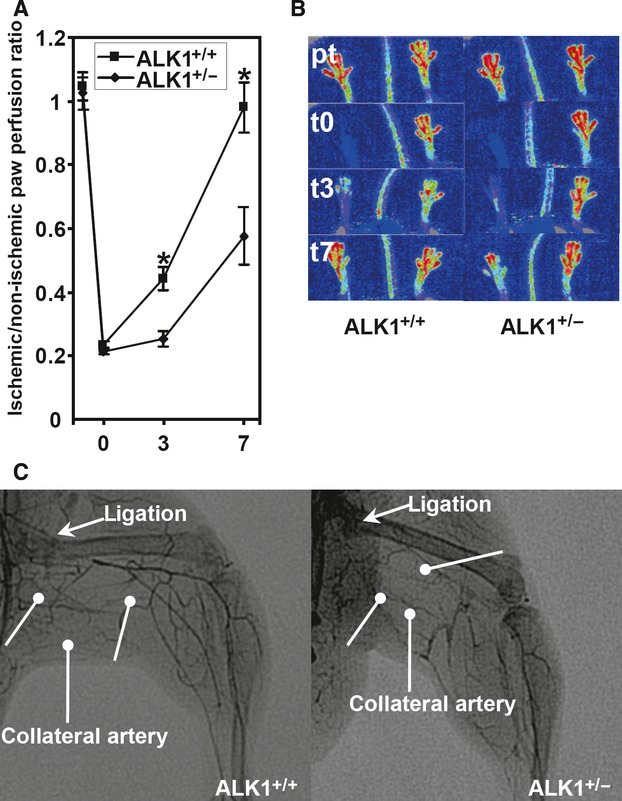
(A) Ischaemic/non-ischaemic paw perfusion ratios of ALK1^+/−^ (*n* = 10) and control mice (*n* = 10). (*P-value <0.01) measured by LDPI over 7 days. (B) Representatives of Laser Doppler Perfusion Imaging (LDPI) images of Eng^+/−^ and control mice that were used to obtain post-mortem angiographic images. Several time-points are displayed: pre-treatment (pt), *t* = 0, *t* = 3 and *t* = 7. (C) Representative angiographic images of upper hind limb of ALK1^+/−^ and control mice 7 days after femoral artery ligation. Arrow indicates site of femoral artery ligation. Rods indicate collateral arteries filled with contrast agent.

To demonstrate the presence of collateral arteries in the adductor thigh muscle, post-mortem angiograms were made 7 days after femoral artery ligation in ALK1^+/−^ mice (*n* = 10) and controls (*n* = 10). LDPI follow-up again demonstrated delayed blood flow recovery in ALK1^+/−^ mice at both 3 and 7 days after induction of hind limb ischaemia, as shown by the reduced ratio of hind limb perfusion (Fig. 6A) and representative LDPI images (Fig. 6B) at the several time-points up to 7 days. And this was similar to our previous observation (Fig. 5). As shown in representative angiograms (Fig. 6C), no prominent differences in collateral artery presence between ALK1^+/−^ and control mice were observed. A Masson Trichrome staining on challenged left adductor thigh muscles, 7 days after femoral artery ligation, revealed abundant collagen deposition. Furthermore, extravasation of erythrocytes was observed in ALK1 heterozygous mice when compared with controls (Supplemental Fig. S1B).

**Fig 7:**
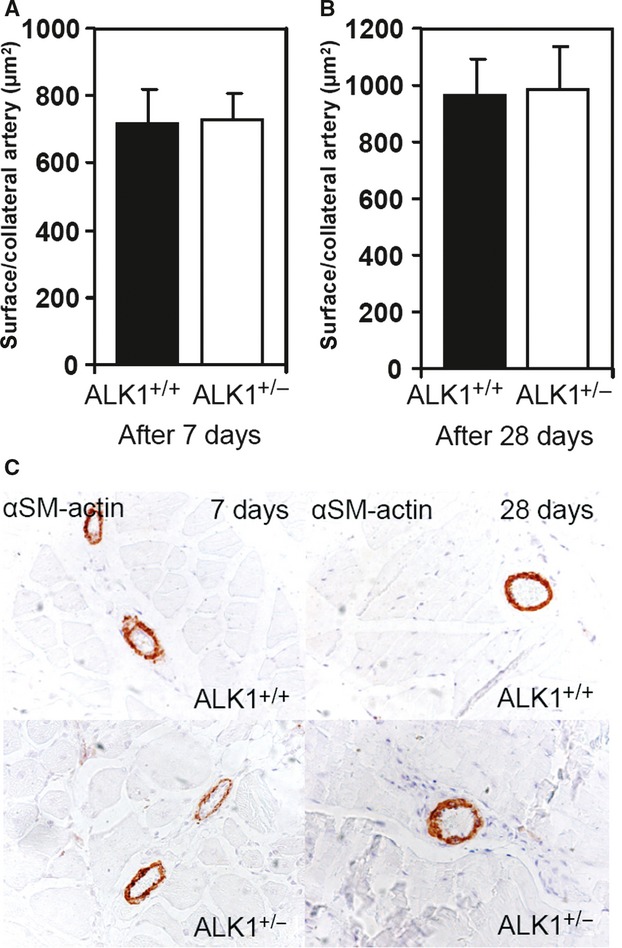
Analysis for collateral artery size, measured as surface (μm^2^) per collateral artery in left adductor thigh muscles of ALK1^+/−^ (open bars) and control mice (closed bars) (*n* = 6–10), (A) 7 days and (B) 28 days after femoral artery ligation. (C) Representative pictures (magnification 15×) of SMC-a stained adductor thigh muscles of ALK1^+/−^ and control mice at 7 and 28 days after femoral artery ligation.

The delay in blood flow recovery in the ALK1^+/−^ mice may also be because of differences in the diameter of the newly formed collaterals. Therefore, the collateral artery size was quantified in ALK1^+/−^ mice by performing an αSM-actin staining for SMCs surrounding the newly formed collaterals in the adductor thigh muscles collected 7 days after ligation. This analysis revealed no significant differences in collateral artery size between ALK1^+/−^ mice when compared with controls (Fig. 7A). Additional analysis for collateral artery size on adductor thigh muscles collected 28 days after ligation also did not reveal differences in collateral artery size between ALK1^+/−^ and controls (Fig. 7B), as can also be appreciated from the representative pictures of SMC-a stained adductor thigh muscles at both time-points in Fig. 7C.

### ALK1^+/−^ mice have larger capillaries after induction of ischaemia

**Fig 8:**
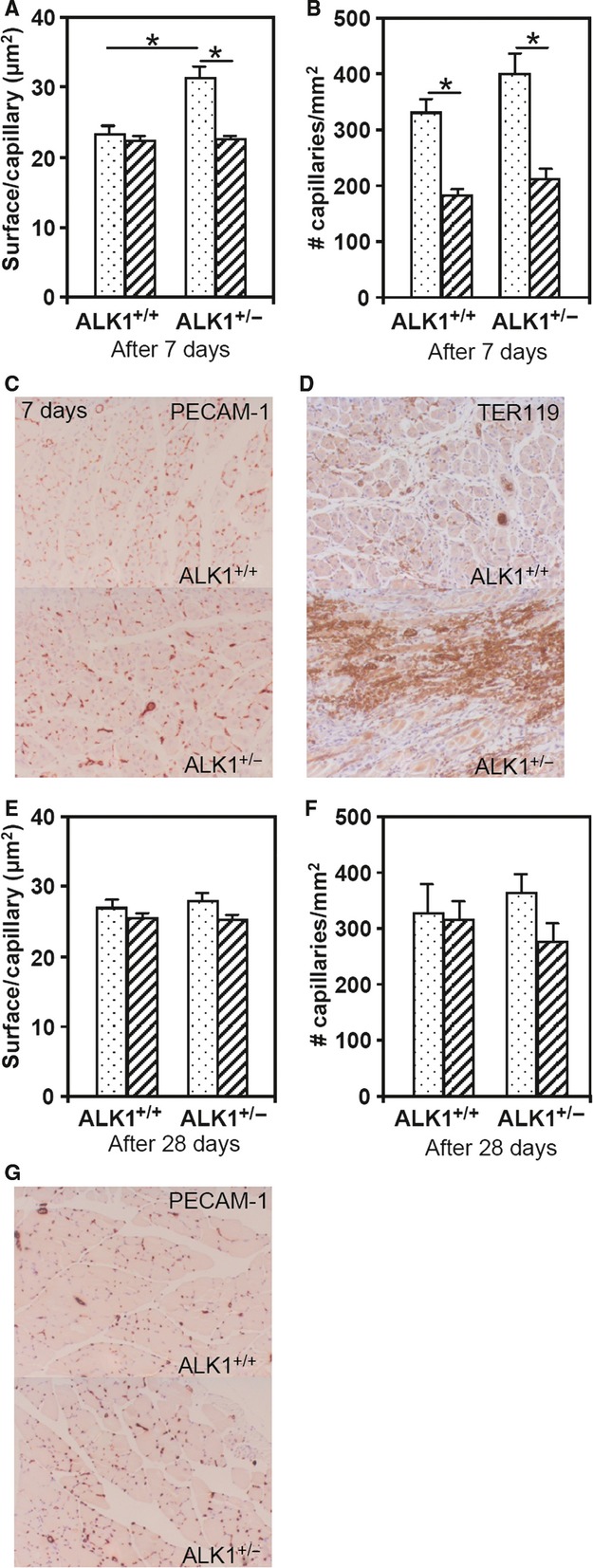
Quantification of ischaemia induced angiogenesis by analysis of PECAM-1 stained left (ligated-side) and right (control-leg) calf muscles of ALK^+/−^ and control mice at 7 and 28 days after femoral artery ligation. Dotted bars represent left calf muscles, dashed bars represent right calf muscles. (*P-value <0.01) (A) Capillary size, measured as surface per individual capillary (μm^2^), 7 days after femoral artery ligation (*n* = 10 per group). (B) Capillary density, measured as number of capillaries per mm^2^, 7 days after femoral artery ligation. (*n* = 10 per group). (C) Representative pictures (magnification 20×) of PECAM-1 stained ischaemic calf muscles of ALK1^+/−^ and control mice at 7 days after femoral artery ligation. (D) Representative pictures (magnification 20×) of TER-119 staining (erythrocytes) demonstrating substantial evasion of erythrocytes outside of the capillary vasculature into the interstitial tissue compartment in ALK1^+/−^ ischaemic calf muscles when compared with ischaemic calf muscles of control mice 7 days after femoral artery ligation. (E) Capillary size, measured as surface per individual capillary (μm^2^), 28 days after femoral artery ligation (*n* = 9–10 per group). (F) Capillary density, measured as number of capillaries per mm^2^, 28 days after femoral artery ligation (*n* = 9–10 per group). (G) Representative pictures (magnification 20×) of PECAM-1 stained ischaemic calf muscles of ALK1^+/−^ and control mice at 28 days after femoral artery ligation.

Blood flow recovery in ALK1^+/−^ mice could be delayed by the newly formed capillary bed in the calf musculature because of differences in the angiogenesis response. Therefore, the angiogenic response was quantified by performing a staining for endothelial cell (using an anti-PECAM-1 antibody) on tissue samples of challenged (left) and unchallenged (right) calf muscles of ALK1^+/−^ and control mice, collected 7 and 28 days after femoral artery ligation. Remarkably, analysis for capillary size revealed significant larger capillaries in ALK1^+/−^ mice compared with controls, 7 days after ligation (Fig. 8A). As depicted in Fig. 8B, our analysis for capillary density did not reveal differences between ALK1^+/−^ and control mice 7 days after ligation, which is also shown by the representative images (Fig. 8C). Induction of angiogenesis was normal in ALK1^+/−^ mice, as a similar significant increase in capillary density was observed in left calf muscles compared with capillary density in right calf muscles, 1.8 and 1.9-fold increase respectively. However, a TER-119 staining for erythrocytes demonstrated erythrocytes in the interstitial tissue compartment that have extravasated from the capillary vasculature (Fig. 8D), a feature observed in 7 of 10 ALK1^+/−^ mice in this group, whereas only one in the controls erythrocyte extravasation could be observed.

At 28 days the average capillary size in left calf muscles of ALK1^+/−^ mice was slightly decreased and not different anymore from capillary size of controls (Fig. 8E). At this time-point, the capillary density in both groups was not further enhanced (Fig. 8F and G). These observations correspond to the LDPI blood flow recovery data as no differences in blood flow recovery existed 28 days after femoral artery ligation.

### Shear stress induced up-regulation of endoglin but not of ALK1 mRNA

**Fig 9:**
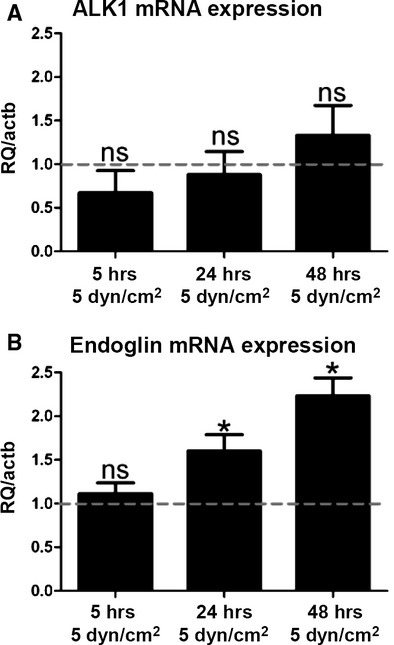
Shear induced regulation of endoglin and ALK1 expression in mouse embryonic endothelial cells (MEEC). Real-time Q-PCR determined messenger RNA (mRNA) levels, normalized for actb (*n* = 4) (A) mRNA levels of ALK1 in MEEC exposed to shear stress (5 dyn/cm^2^) for 5, 24 and 48 hrs. The dashed line represents the expression level of ALK1 mRNA in MEEC under static condition (0.5 Pa) and is indexed at 1.0, all time-points are compared with the static condition mRNA expression. (*P*-values: 5 hrs *P* 0.44; 24 hrs *P* 0.61; 48 hrs *P* 0.32). (B) mRNA levels of endoglin in MEEC exposed to shear stress (5 dyn/cm^2^) for 5, 24 and 48 hrs. The dashed line represents the expression level of endoglin mRNA in MEEC under static condition (0.5 Pa) and is indexed at 1.0, all time-points are compared with the static condition mRNA expression. (*P-value <0.05; *P*-values: 5 hrs *P* 0.36; 24 hrs *P* 0.04; 48 hrs *P* 0.003).

The results of these experiments show a striking difference in response in the Eng^+/−^ and ALK1^+/−^ mice with regard to the arteriogenic response. As collateral formation is a shear stress induced process, the observed differences might be because of different responses of endoglin and ALK1 to shear stress. To study this hypothesis in more detail, murine embryonic endothelial cells (MEEC) were cultured under condition of flow, to mimic shear stress *in vitro*, for a period of 5, 24 and 48 hrs and subsequently the cells were harvested and the expression of endoglin and ALK1 was analysed at the mRNA level. As can be observed in Fig. 9A and B, no differences in both ALK1 and endoglin mRNA expression in MEEC were observed after 5 hrs under flow conditions when compared with static conditions, whereas a significant 50% and 100% increase in endoglin mRNA expression was observed in the MEEC exposed to a flow of 5 dyn/cm^2^ for 24 hrs and 48 hrs respectively. These data clearly demonstrate a difference in shear stress sensitivity for endoglin and ALK1 expression in ECs.

## Discussion

In this study, we addressed the role of TGF-β signalling receptors endoglin and ALK1 in blood flow recovery after induction of acute hind limb ischaemia in mice. Blood flow recovery is depending on both the recruitment of pre-existing arterioles to become collateral arteries, a shear stress driven process, as well as angiogenic sprouting of capillaries into the ischaemic tissue, a hypoxia driven process. We demonstrated that haplo-insufficiency for endoglin affects both shear induced collateral artery growth and ischaemia induced capillary angiogenesis, whereas haplo-insufficiency for ALK1 only resulted in disturbed capillary angiogenesis by the formation of dysplastic capillaries. *In vitro* data of mouse embryonic ECs exposed to shear stress support this additional contributory role of endoglin in shear stress induced collateral artery growth, as increased levels of endoglin messenger RNA (mRNA) but not of ALK1 were detected in these cells.

A previous study [32] demonstrated reduced vascularity in adductor thigh muscles of endoglin heterozygous mice after hind limb ischaemia, which resembles to our observation of reduced vascularity in this study. Moreover, in our hind limb ischaemia model, we studied blood flow recovery in both the upper and lower hind limb, which allowed us to discriminate between shear induced collateral artery growth and ischaemia induced capillary angiogenesis in the same mouse model. By this approach we were able to demonstrate that ALK1 and endoglin contribute differently to these two processes of blood flow recovery. We also observed that injury changed endoglin expression in blood vessels in challenged adductor thigh muscles. After hind limb ischaemia, endoglin heterozygous mice displayed significant reduced collateral artery size, indicating disturbed arteriogenesis when compared with littermate control mice. Furthermore, ischaemia induced angiogenesis in the calf muscles of these mice resulted in disturbed capillary formation, represented by significant larger capillaries than in calf muscles of controls. Unlike previously reported reduced proliferation of endoglin heterozygous ECs by Jerkic *et al*. [32], proliferation did not seem to be affected in our study based on the results of a Ki67 proliferation staining. No differences in Ki67 positive cells were observed between endoglin heterozygous and control mice, 7 days after ischaemia (Supplemental Fig. S3A). Together with our observation that capillary density was not affected in the calf muscles of endoglin heterozygous mice, suggests that endothelial cell migration and re-organization and not growth are the disturbed angiogenic processes in these mice. Mahmoud *et al*. also postulated that endoglin plays a part in restricting endothelial cell proliferation and that capillary remodelling is impaired in conditional endoglin knockout mice by a defect in intercalation of ECs during capillary tube formation [38], which gives rise to small opportunistic shunts once blood flow commences [39]. Capillary dysfunction in our Eng^+/−^ mice was indicated by staining for erythrocytes, which demonstrated evasion of erythrocytes outside of the capillary vasculature into the interstitial tissue compartment. The fact that both shear induced arteriogenesis and ischaemia induced angiogenesis are affected in the endoglin heterozygous mice, suits well with the fact that endoglin is expressed by both endothelial and vascular SMCs [16, 40] and by fibroblasts in the perivascular stroma of arteries [30].

In ALK1 heterozygous mice blood flow recovery was found to be delayed by only defective angiogenesis but not shear induced collateral artery growth, as these mice only displayed significant larger capillaries indicating capillary dysplasia [10, 12]. It has been described that ALK1 heterozygous mice stimulated with angiogenic growth factors have dysplastic and dilated capillaries and arteriovenous malformations [41]. Our hind limb ischaemia model also induces the production of angiogenic growth factors [35], which explains significant increase in capillary size 7 days after ischaemia. A Ki67 proliferation staining on calf muscles 7 days after ischaemia revealed low numbers of Ki67 positive cells and no differences were observed between ALK1 heterozygous mice and controls (Supplemental Fig. S3B). This indicates that endothelial cell proliferation was not affected in these mice, as a significant increase in capillary density, similar to controls, was observed. Here, analysis for erythrocytes outside capillary vessel walls also indicated that these dysplastic capillaries were indeed leaky and this implicates reduced functionality [10, 42], which should have had consequences for blood flow recovery in these mice. The significant increase in capillary size and densities in non-ischaemic legs of the ALK1 littermate controls between 7 and 28 days after ligation is most probably a systemic effect of increased levels of circulating pro-angiogenic factors that are produced in the ischaemic leg.

Although both the endoglin and ALK1 strains are bred on a C57BL/6 background, the littermate controls from these two strains both display a reduced response compared with the original C57BL/6 background strain. This is likely caused by the inbred nature of these strains. The degree of inbred varies, 8 backcrosses for endoglin and 13 for ALK1, which makes direct comparison of vessel sizes between the two strains difficult. Actually, this comparison was not the goal of this study as we wanted to address the individual roles of these two TGF-β receptors. The fact that in ALK1 heterozygous mice only ischaemia induced angiogenesis is impaired but not shear induced collateral artery growth can be explained by the fact that ALK1 is mainly expressed in ECs [15], but not, unlike endoglin [43] in vascular SMCs that play a role in collateral artery remodelling. Although ALK1 is suggested to promote SMC recruitment and differentiation, mainly based on observations in ALK1-null mice, haplo-insufficiency for ALK1 did not result in hampered collateral artery growth in our hind limb ischaemia mouse model. An explanation could be that ALK1 heterozygosity has milder consequences than total depletion of ALK1. Besides this, ALK1 is demonstrated to limit nascent vessel calibre in reaction to shear stress, ALK1 haplo-insufficiency might therefore have facilitated normal collateral artery growth by reduced inhibition [29]. Finally, the observation that shear stress on cultured MEECs induced high messenger RNA (mRNA) levels of endoglin, but not ALK1, provides a body of mechanistic evidence for this *in vivo* observation of impaired shear induced collateral artery formation in endoglin heterozygous mice but not in ALK1 heterozygous mice.

Therefore, we conclude that both TGF-β receptors endoglin and ALK1 play a contributory role in blood flow recovery. Importantly, our study for the first time demonstrates that endoglin is essential in both shear induced collateral artery growth and in ischaemia induced capillary angiogenesis, whereas ALK1 is only involved in ischaemia induced capillary angiogenesis.

## Supporting information

**Fig. S1.** Representative pictures (magnification 20×) of Masson trichrome stained challenged left adductor thigh muscles 7 days after femoral artery ligation. (**A**) Abundant collagen deposition and extravasation of erythrocytes is displayed in muscle of an endoglin heterozygous mouse when compared with muscle of corresponding control mouse. (**B**) Similar to endoglin heterozygous mouse muscle, ALK1 heterozygous mouse muscle displays similar distribution of collagen and erythrocytes, which is abundant when compared with corresponding control mouse muscle.

**Fig. S2.** Representative fluorescence pictures of Endoglin staining 7 days after femoral artery ligation on challenged left adductor thigh muscles and on unchallenged control adductor thigh muscles. DAPI (blue staining) was used to counterstain the nuclei, endoglin was stained using the FITC labelled rabbit polyclonal antiendoglin antibody KRE, green signal (1) (**A**) Endoglin staining is much more pronounced around ECs in the challenged left adductor thigh muscle of the control mouse when compared with the left adductor thigh muscle of the endoglin heterozygous mouse that show reduced fluorescence around ECs, as can be appreciated from the higher magnification inserts. (**B**) The unchallenged adductor thigh muscle of the control legs of the endoglin heterozygous mouse and the control showing very low fluorescence signal for endoglin.

**Fig. S3.** Representative pictures (magnification 20×) of Ki67 stained challenged left adductor thigh muscles of (**A**) Endoglin heterozygous and control mice and of (**B**) ALK1 heterozygous and control mice, 7 days after ligation of the femoral artery. The antibody used was the anti-Ki67 antibody NCL-L-Ki67-MM1, from Novo Castra.
