# Supplementary figures and images for: Shear induced collateral artery growth modulated by endoglin but not by ALK1
Source: J Cell Mol Med. 2012 Sep 26;16(10):2440–50. doi: 10.1111/j.1582-4934.2012.01561.x (PMC3823438; doi:10.1111/j.1582-4934.2012.01561.x)

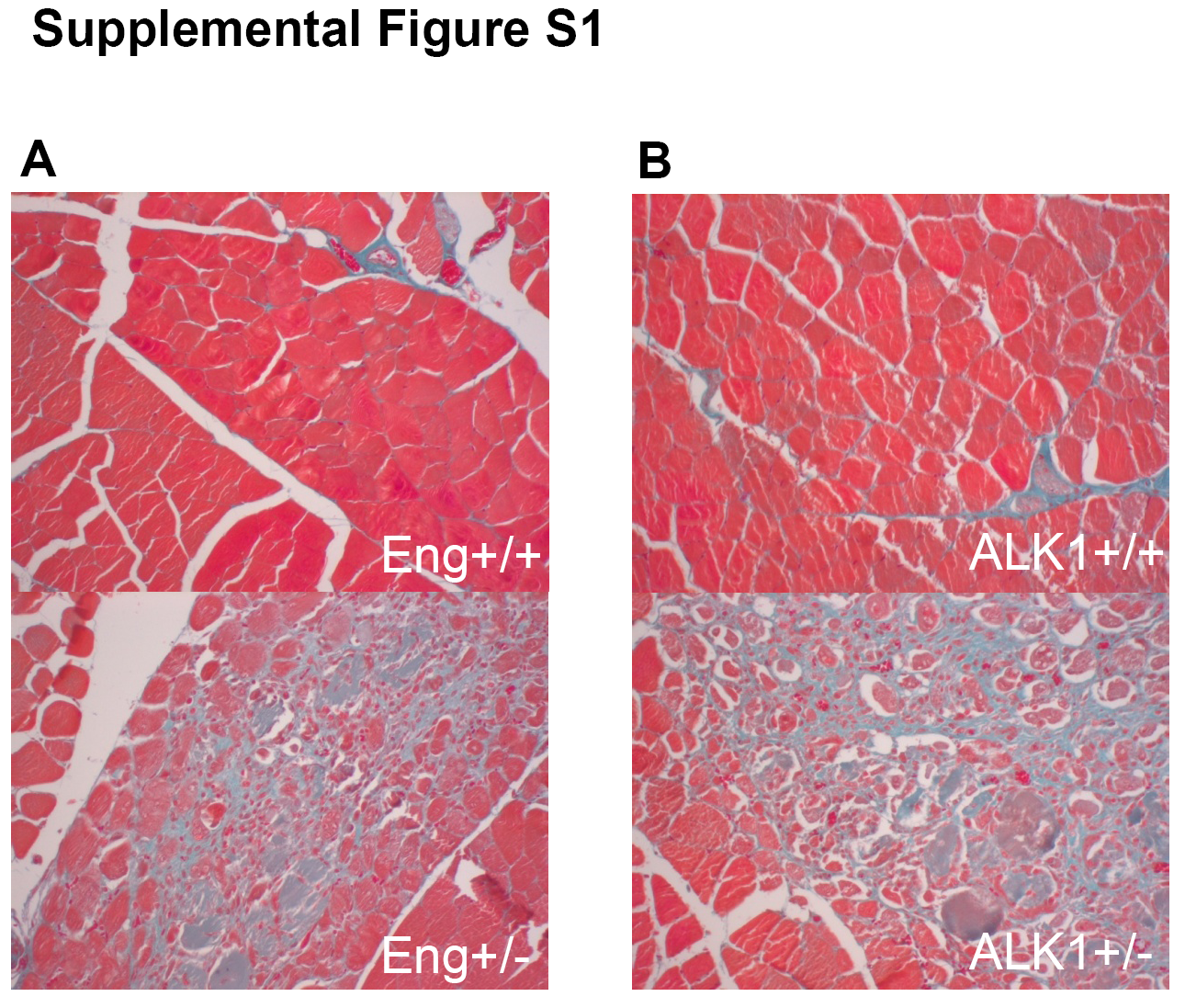

Supplement: Supplementary file 1 [file jcmm0016-2440-SD1.tif]

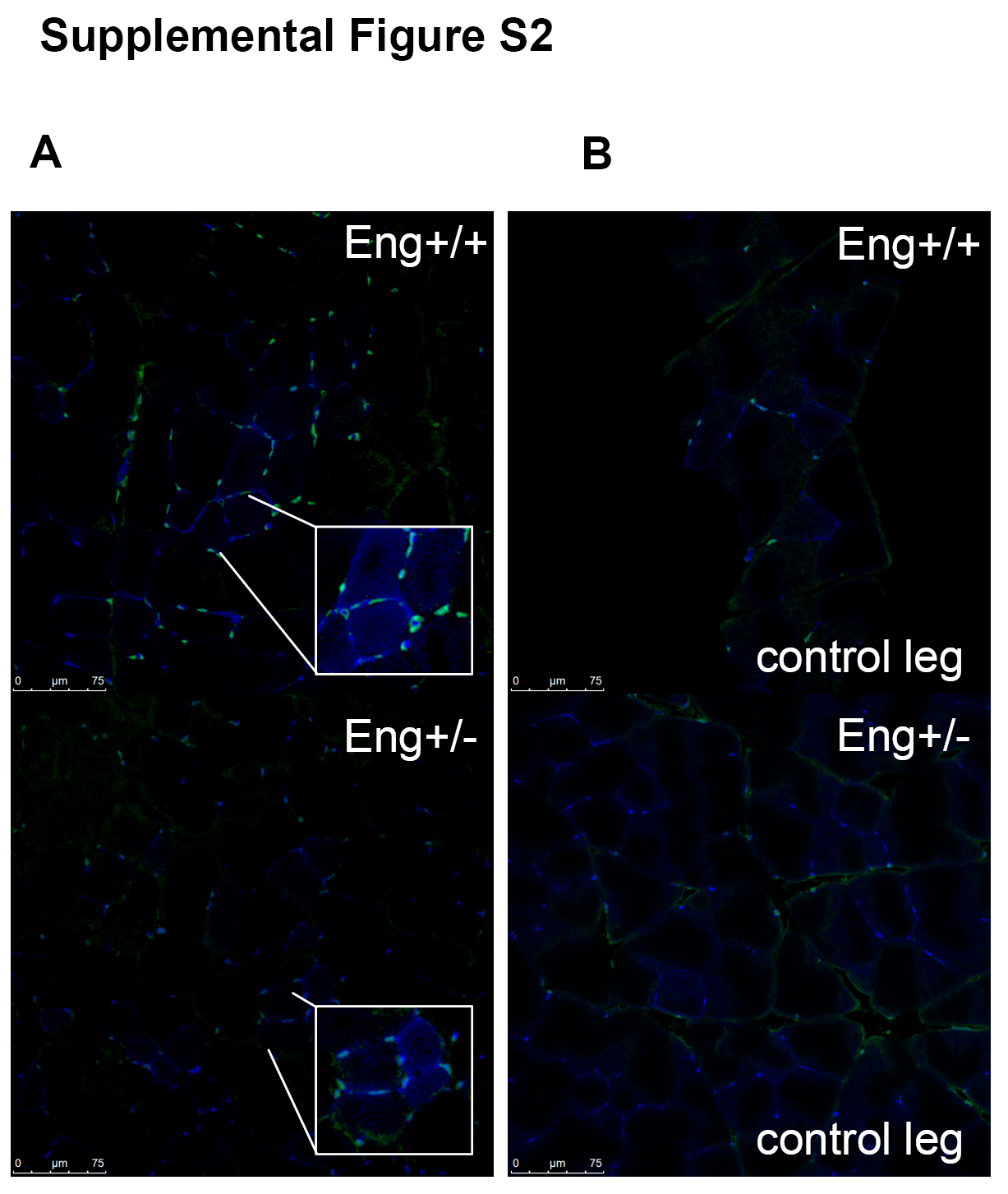

Supplement: Supplementary file 2 [file jcmm0016-2440-SD2.tif]

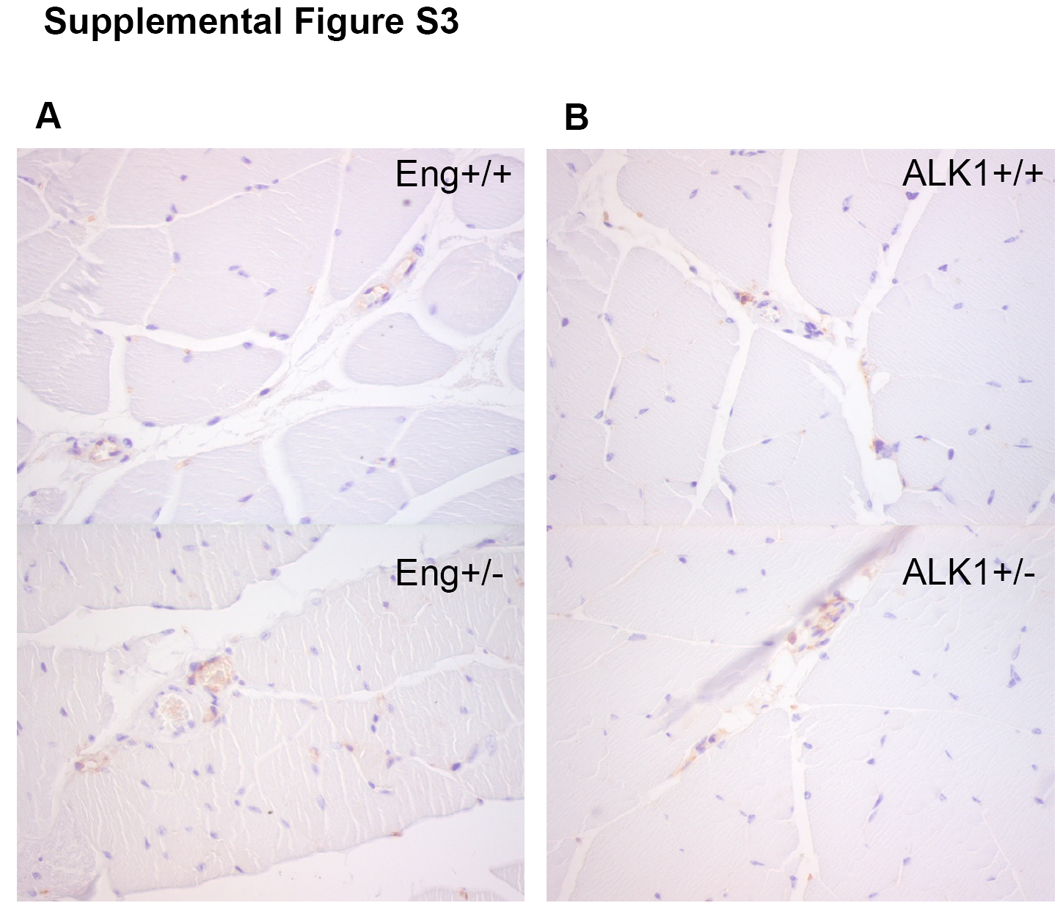

Supplement: Supplementary file 3 [file jcmm0016-2440-SD3.tif]
