# Supplementary material for: Shear induced collateral artery growth modulated by endoglin but not by ALK1
Source: J Cell Mol Med. 2012 Sep 26;16(10):2440–50. doi: 10.1111/j.1582-4934.2012.01561.x (PMC3823438; doi:10.1111/j.1582-4934.2012.01561.x)
Supplement: Supplementary file 4 [file jcmm0016-2440-SD4.doc]

Supplemental data

Supplemental Figure Legends

**Figure S1.** Representative pictures (magnification 20x) of Masson Trichrome stained challenged left adductor thigh muscles 7 days after femoral artery ligation. **A.** Abundant collagen deposition and extravasation of erythrocytes is displayed in muscle of an endoglin heterozygous mouse when compared to muscle of corresponding control mouse. **B.** Similar to endoglin heterozygous mouse muscle, ALK1 heterozygous mouse muscle displays similar distribution of collagen and erythrocytes, which is abundant when compared to corresponding control mouse muscle.

**Figure S2.** Representative fluorescence pictures of Endoglin staining 7 days after femoral artery ligation on challenged left adductor thigh muscles and on unchallenged control adductor thigh muscles. DAPI (blue staining) was used to counterstain the nuclei, endoglin was stained using the FITC labeled rabbit polyclonal anti-endoglin antibody KRE, green signal (1) **A.** Endoglin staining is much more pronounced around endothelial cells in the challenged left adductor thigh muscle of the control mouse, when compared to the left adductor thigh muscle of the endoglin heterozygous mouse that show reduced fluorescence around endothelial cells, as can be appreciated from the higher magnification inserts. **B.** The unchallenged adductor thigh muscle of the control legs of the endoglin heterozygous mouse and the control, showing very low fluorescence signal for endoglin.

**Figure S3.** Representative pictures (magnification 20x) of Ki67 stained challenged left adductor thigh muscles of **A.** Endoglin heterozygous and control mice, and of **B.** ALK1 heterozygous and control mice, 7 days after ligation of the femoral artery. The antibody used was the anti-Ki67 antibody NCL-L-Ki67-MM1, from Novo Castra.

References:

1. Yamashita H, Ichijo H, Grimsby S, Morén A, ten Dijke P, Miyazono K. Endoglin forms a heteromeric complex with the signaling receptors for transforming growth factor-beta. J Biol Chem (1994),269, p 1995-2001.
